# Characterization of an Atypical *Trypanosoma brucei* Hsp70 Demonstrates Its Cytosolic-Nuclear Localization and Modulation by Quercetin and Methylene Blue

**DOI:** 10.3390/ijms22136776

**Published:** 2021-06-24

**Authors:** Adélle Burger, Paula Macucule-Tinga, Stephen John Bentley, Michael Hans Ludewig, Ndumiso Nhlakanipho Mhlongo, Addmore Shonhai, Aileen Boshoff

**Affiliations:** 1 Department of Biochemistry, University of Venda, Private Bag X5050, Thohoyandou 0950, South Africa; Addmore.Shonhai@univen.ac.za; 2 Biotechnology Innovation Centre, Faculty of Science, Rhodes University, PO Box 94, Makhanda/Grahamstown 6140, South Africa; Paulamacucule@gmail.com (P.M.-T.); Stephenjohnbentley@gmail.com (S.J.B.); M.Ludewig@ru.ac.za (M.H.L.); 3 Department of Medical Biochemistry, University of KwaZulu-Natal, Private Bag X54001, Durban 4000, South Africa; MhlongoN4@ukzn.ac.za

**Keywords:** *Trypanosoma brucei*, chaperone, TbHsp70.c, TbHsp70, TbSTi1, Tbj2, ATPase activity, quercetin, methylene blue

## Abstract

*Trypanosoma brucei* (Tb) harbours twelve Hsp70 chaperones. Of these, four are predicted to reside in the parasite cytosol. TbHsp70.c is predicted to be cytosolic and upregulated upon heat stress and is an ATPase that exhibits holdase chaperone function. Cytosol-localized Tbj2 stimulates the ATPase activity of TbHsp70.c. In the current study, immunofluorescence confirmed that TbHsp70.c is both a cytosolic and a nuclear protein. Furthermore, in silico analysis was used to elucidate an atypical linker and hydrophobic pocket. Tellingly, TbHsp70.c lacks the EEVD and GGMP motifs, both of which are implicated in substrate selectivity and co-chaperone binding in canonical Hsp70s. Far western analysis revealed that TbSTi1 interacts directly with TbHsp70 and TbHsp70.4, but does not bind TbHsp70.c. We further investigated the effect of quercetin and methylene blue on the Tbj2-driven ATPase activity of TbHsp70.c. We established that quercetin inhibited, whilst methylene blue enhanced, the Tbj2-stimulated ATPase activity of TbHsp70.c. Furthermore, these inhibitors were lethal to parasites. Lastly, we used molecular docking to show that quercetin and methylene blue may bind the nucleotide binding pocket of TbHsp70.c. Our findings suggest that small molecule inhibitors that target TbHsp70.c could be developed to serve as possible drug candidates against *T. brucei*.

## 1. Introduction

African sleeping sickness is a parasitic disease that threatens millions of people in sub-Saharan Africa. The etiological agent of this potentially fatal disease is a protozoan parasite *Trypanosoma brucei* (*T. brucei*), which is endemic to 36 countries [1]. The people most at risk of contracting sleeping sickness (human African trypanosomiasis) are from the rural populations that are dependent on fishing, animal husbandry, agriculture, and hunting [1]. There remains a necessity to develop effective therapeutic strategies against African trypanosomiasis due to factors such as increasing parasite resistance, treatment toxicity, and, lastly, complexity of diagnosing and treating the disease [2]. The parasite is transmitted periodically between its mammalian host and an insect vector, the tsetse fly. Thus, this parasite switches between mammalian blood and tissue fluids, and insect alimentary gut and salivary glands. As the parasite differentiates between the bloodstream and procyclic forms, it needs to adapt to alterations in temperature, pH, nutrients, and oxygen tension in order to survive. Some of these stress factors could alter the prototypical genetic expression of proteins specifically participating in homeostasis, thereby affecting survival, reproduction, and transmission of the parasite. Genes involved in growth are automatically downregulated and proteins associated with cellular survival, such as heat shock proteins, are upregulated [3]. Chaperones upregulated under these stress conditions are involved in the maintenance of proteostasis, including protecting hydrophobic surfaces exposed in misfolded proteins and during protein refolding, and they are associated with the ubiquitin proteasome system and autophagy to facilitate the removal of terminally misfolded polypeptides [4,5]. Heat shock proteins present prospective antiparasitic drug targets [6,7]. The heat shock protein 70 (Hsp70) family are involved in cell survival, signalling, and maintaining proteostasis under normal conditions and in response to cellular stress, highlighting their importance as critical molecular chaperones [8,9]. Hsp70s have been implicated in immunomodulation in various chronic inflammatory diseases and in cancer [10]. Parasitic Hsp70s are well established in their primary role as facilitators of protein folding. In addition, they regulate protein translocation and proteolytic degradation of misfolded and aggregated proteins [11,12,13].

Members of the ubiquitously expressed Hsp70 family are well conserved. However, despite their high levels of conservation, there is evidence of functional specificity amongst the Hsp70s [12,13,14]. Structurally, Hsp70 is composed of two domains, the N-terminal nucleotide binding domain (NBD) and the C-terminal substrate binding domain (SBD). The well conserved NBD binds and hydrolyses ATP to ADP [15]. The more divergent SBD is composed of α- and β-subdomains; the α-helical subdomain at the C-terminus functions as a lid, folding over misfolded substrate which binds to the β-subdomain [16]. Nucleotides regulate Hsp70 function in that Hsp70 displays an increased affinity for substrate in its ADP-bound state, which is released upon binding ATP. The NBD and SBD are joined together by a highly conserved linker. The linker is not only involved in regulating the conformation of Hsp70, but also facilitates co-chaperone binding and modulates the affinity of Hsp70 for misfolded substrate [17]. Despite the high degree of conservation, there are unique features amongst the Hsp70s. One of these features are the GGMP repeat residues, which have been reported to modulate the functional specificity of a plasmodial Hsp70, PfHsp70-1, by facilitating substrate and co-chaperone binding [17]. GGMP repeats are reportedly abundant in the cytosolic Hsp70s of parasitic origins, but are missing or under-represented in other organisms [17].

Hsp70 activity is driven by co-chaperones. Hsp40s, also called J-proteins, function as co-chaperones of Hsp70s. J-proteins stimulate the ATPase activity of Hsp70s by binding to their NBDs, and they also present misfolded substrates to Hsp70 for refolding [18]. Hsp70-Hsp90 organizing protein (Hop)/stress inducible protein 1 (STi1) is another distinct co-chaperone of Hsp70. Hop/STi1 functions as a scaffold protein, linking Hsp70 and Hsp90 via the C-terminal EEVD motif, such that substrates that require further folding may be passed from Hsp70 to Hsp90 [19,20]. Hop/STi1 forms a ternary complex with Hsp70 and Hsp90 using its tetratricopeptide repeat (TPR) domains that primarily bind the C-terminal EEVD and MEEVD motifs of Hsp70 and Hsp90, respectively [19,20].

The *T. brucei* genome encodes 12 members of the Hsp70 family, four of which are predicted to localize in the cytosol, and 67 putative J proteins, of which five are type I J-proteins [21]. Of significance to this study is an atypical trypanosomal Hsp70, TbHsp70.c. In silico analysis revealed TbHsp70.c to be an eukaryotic isoform exhibiting novel features as it did not cluster phylogenetically with any of the other primary Hsp70 proteins [22]. TbHsp70.c was shown to be upregulated under heat stress, and exhibited holdase function as it suppressed thermally induced aggregation of a model protein in vitro [22]. Furthermore, a type I J-protein, Tbj2, was reported to enhance the ATPase activity of TbHsp70.c, suggesting its role as a potential co-chaperone of TbHsp70.c. TbHsp70.c is predicted to localize in the cytosol [23], and also localizes to the nucleoplasm [24]. Tbj2 and TbSTi1 are resident in the cytoplasm [25]. A high-throughput phenotypic study was conducted by knocking down *T. brucei* genes using RNAi [26]. Silencing certain members of the Hsp70 and J-protein families resulted in loss of fitness at various stages of the parasite’s lifecycle [26], highlighting their importance in protozoal biology. Tbj2 is essential to the survival of the parasite [25,26].

In the current study, we investigated the subcellular localization of TbHsp70.c in the bloodstream form of the parasite and our data suggest a cytosolic localization in addition to nuclear localization. We sought to highlight the unique features of TbHsp70.c through multiple sequence alignments and homology modelling. The in silico analysis revealed a well conserved NBD, typical of canonical Hsp70s. The linker sequence of TbHsp70.c showed divergence in the first subsection as it lacks charged residues, as opposed to the presence of charged residues in the first subsection of canonical Hsp70s. TbHsp70.c possesses select unique residues implicated in direct substrate binding. Furthermore, both the GGMP repeat residues and the EEVD motif are absent in TbHsp70.c. Our findings established that TbHsp70.c is incapable of binding TbSTi1. We further performed biochemical assays to investigate the effect of methylene blue and quercetin on the chaperone activity of TbHsp70.c. We discuss the significance of the atypical features of TbHsp70.c on its essential role in the development of *T. brucei* and its prospects as a druggable candidate.

## 2. Results

### 2.1. Immunofluorescence Suggests That TbHsp70.c Localizes in Both the Cytosol and Nucleus

**Figure 1:**
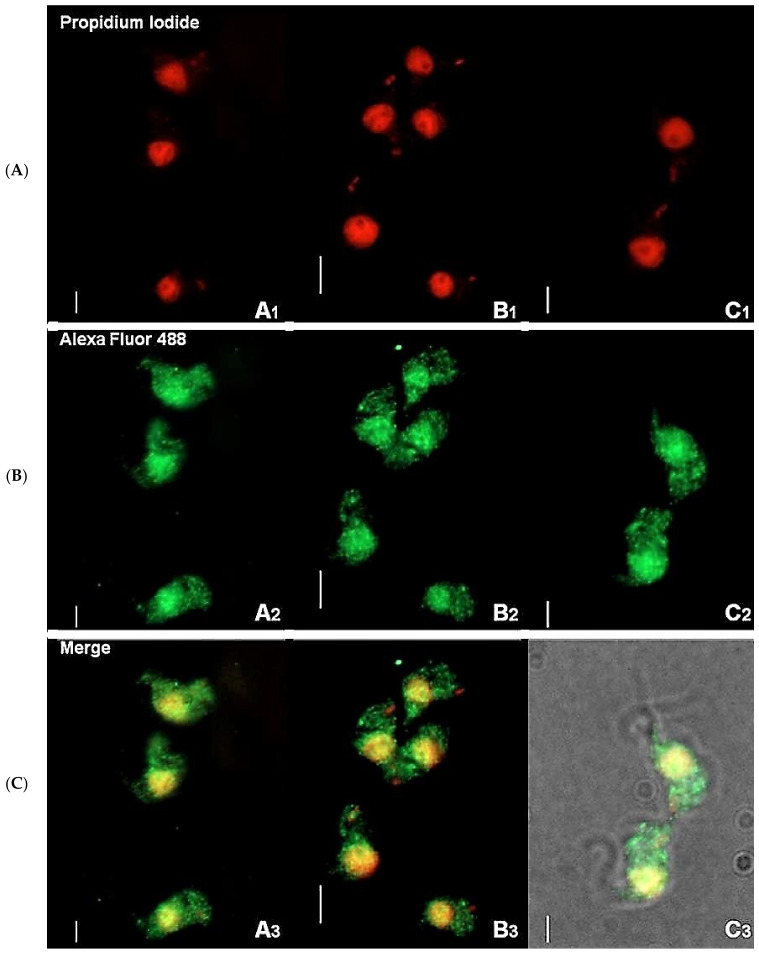
Immunofluorescence staining of *T. brucei* bloodstream form suggests cytosolic localization of TbHsp70.c. Distribution of TbHsp70.c in *T. b. brucei* cells using TbHsp70.c primary antibody, and Alexa Fluor 488 secondary antibody (**A**–**C**). Parasite DNA was stained with propidium iodide. Upright scale bar, 5 µm. Rows: Propidium iodide—parasite DNA, including the nucleus and kinetoplast detected with a UV filter, shown in red; Alexa Fluor 488—localization of TbHsp70.c; Merge—amalgamated image of DNA staining and TbHsp70.c localization.

**Figure 2:**
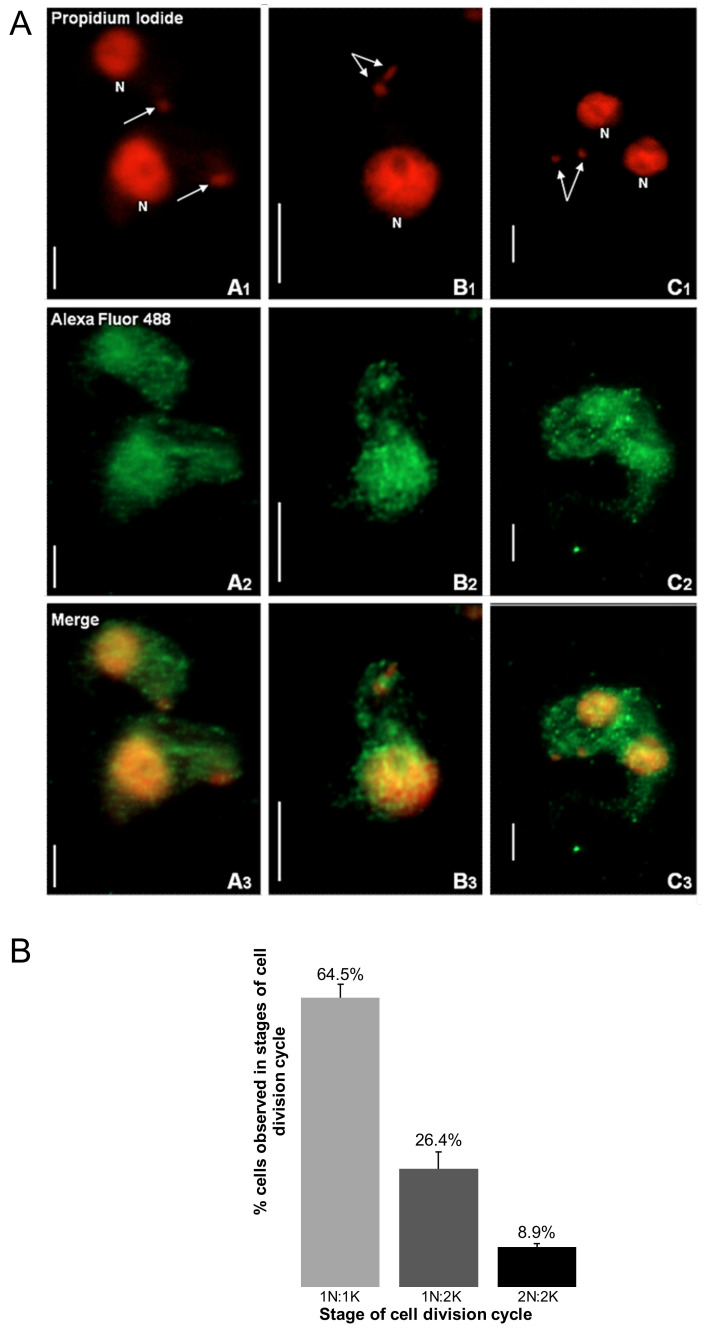
The distribution of TbHsp70.c is cytosolic and nucleic along the *T. brucei* life cycle. (**A**) Morphology of the kinetoplast at different stages of the *T. brucei* bloodstream form cell cycle. The top row represents the propidium iodide signal in enlarged images of the number and location of nuclei (N) and kinetoplasts (arrow). Upright scale bar, 5 µm. Columns: A—cells have 1 nucleus and 1 kinetoplast (1N:1K); B—1N:2K; C—2N:2K. Rows: Propidium iodide—parasite DNA detected with a UV filter, shown in red; Alexa Fluor 488—localization of TbHsp70.c; Merge—amalgamated image of DNA staining and TbHsp70.c localization. (**B**) The percentage *T. b. brucei* cells counted containing 1N:1K, 1N:2K and 2N:2K. Error bars represent the standard deviation between two replicates.

Immunofluorescence-based data showed that TbHsp70.c is cytosolic and nucleic at the bloodstream form of the parasite (Figure 1). This finding is consistent with a previous study showing that TbHsp70.c is localized in the nucleoplasm [24]. Typically, differentiating trypanosomes possess a nuclei (N) to kinetoplast (K) ratio of 1N:1K which progresses to 2N:2K. This phenomenon was evident in the current study (Figure 2). The apparent cytosolic and nucleic localization of TbHsp70.c did not appear to be influenced by the different stages of the cell cycle (Figure 2).

### 2.2. TbHsp70.c Possesses Unique Residues in the Hydrophobic Pocket, and Lacks Some Key Motifs

**Figure 3:**
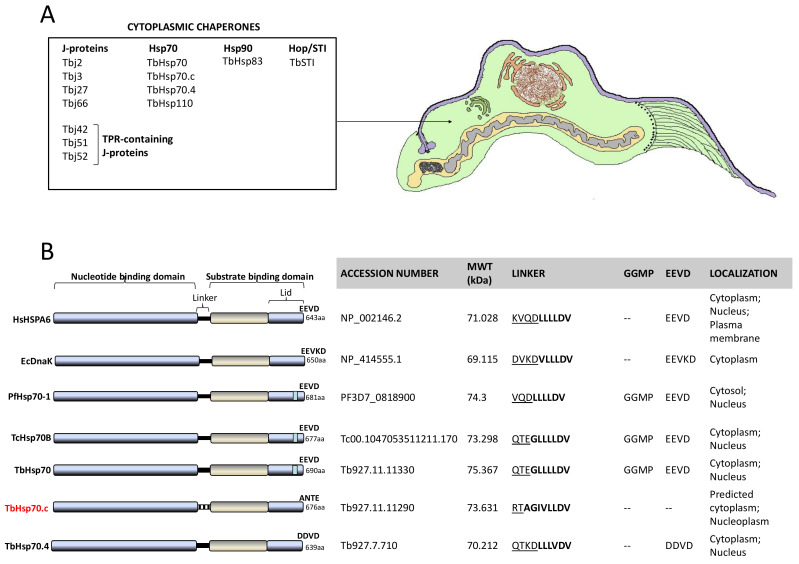
Cytosolic heat shock proteins of *T. brucei* and distinct features of TbHsp70.c. (**A**) Schematic showing organelles and structures of a bloodstream form trypanosome as would be observed in thin sections by transmission electron microscopy. (**B**) Features of canonical Hsp70s were compared to highlight distinct features of TbHsp70.c based on multiple sequence alignments [13,17]. Underlined residues represent the hydrophilic region and residues in bold represent the hydrophobic regions of the linker.

A multiple sequence alignment of TbHsp70.c relative to its homologues and orthologues is shown (Figure S1). Residues previously determined to be essential for *Escherichia coli* (*E. coli*) DnaK function, such as the proline switch [27], and those implicated in interdomain communication are conserved across all the species (Figure S1). Residues of TbHsp70.c that directly associate with J proteins are conserved (Figure S1). The linker region of TbHsp70.c is divergent to that of the well characterized eukaryotic and prokaryotic Hsp70s (Figure S1). The following residues represent the linker of TbHsp70.c: RTAGIVLLDV in contrast to the canonical (DVKDVLLLDV) linker sequence of *E. coli* DnaK. Notably, the first four residues (RTAG) of TbHsp70.c are distinct (uncharged) in contrast to those of *E. coli* DnaK (DVKD), which are charged. The function of Hsp70 is regulated by allostery through nucleotide binding at the NBD, which influences Hsp70’s affinity for substrate [17]. The linker of Hsp70 facilitates its allosteric function. This implies that TbHsp70.c could undergo allostery in a unique fashion, in comparison to canonical Hsp70s (Figure 3) [17]. Notably, the GGMP repeats are present in TbHsp70 and TcHsp70B. However, they are absent in both TbHsp70.c and TbHsp70.4 (Figure 3B). It is thus not entirely surprising that the highly conserved EEVD motif, which is required for interaction of Hsp70 with its co-chaperone, STi1, is absent from TbHsp70.c (Figure 3B). It has been established that both the EEVD motif and the GGMP repeat motif are implicated in substrate selectivity and co-chaperone binding [13]. The presence of various unique features in TbHsp70.c suggests that it is an atypical Hsp70.

### 2.3. Homology Modelling of the TbHsp70.c Substrate Binding Domain Highlights a Unique Hydrophobic Pocket

**Figure 4:**
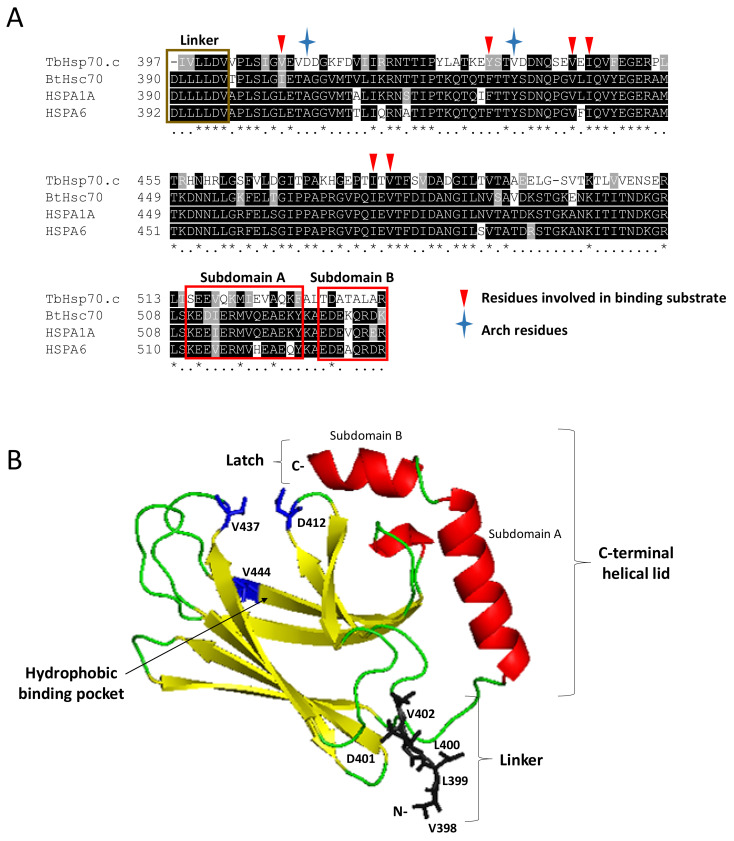
Identification of key residues of TbHsp70.c and their predicted orientation in space. A multiple sequence alignment of the SBD of TbHsp70.c, bovine Hsc70, human HSPA1A and HSPA6 (**A**). Segments highlighted in yellow and red represent predicted secondary structures β-sheets and α-helices, respectively, and coincide with the modelled structure in panel B. Asterisk (*) symbols indicate positions that have fully conserved residues, and period (.) symbols indicate conservation between groups of weakly similar properties. Ribbon representation of the homology model of TbHsp70.c (**B**). The predicted homology model of TbHsp70.c (residues 397–540) highlights the linker region in brown; the predicted secondary structure β-sheets in yellow; predicted α-helices in red; arch and one pocket residue in blue. The root mean square deviation (RMSD) was calculated to be 0.257Å. PyMol was used to visualize the model [29].

The multiple sequence alignment of TbHsp70.c with canonical Hsp70s highlights the arch residues (D412 and V437) and several other residues located in its substrate binding cavity (V409, Y434, V444, I446, I479 and V481) that bind substrate directly. Notably, D412 and V437 of TbHsp70.c substitute for the arch residues A406 and Y431 that occur in HSPA1A and Hsc70 (Figure 4A) [28]. Furthermore, V409 and Y434 of TbHsp70.c substitute for residues L403 and F428 of HSPA1A and Hsc70, which bind substrate directly (Figure 4A) [28]. Residues V444, I446, I479, and V481 of TbHsp70.c, implicated in binding substrate directly, are conserved, highlighting their importance in the function of this chaperone (Figure 4) [28]. A homology model of TbHsp70.c further highlights the positioning in space of these atypical acidic residues located in its substrate binding domain (Figure 4B).

### 2.4. TbHsp70C Does Not Bind TbSTi1

**Figure 5:**
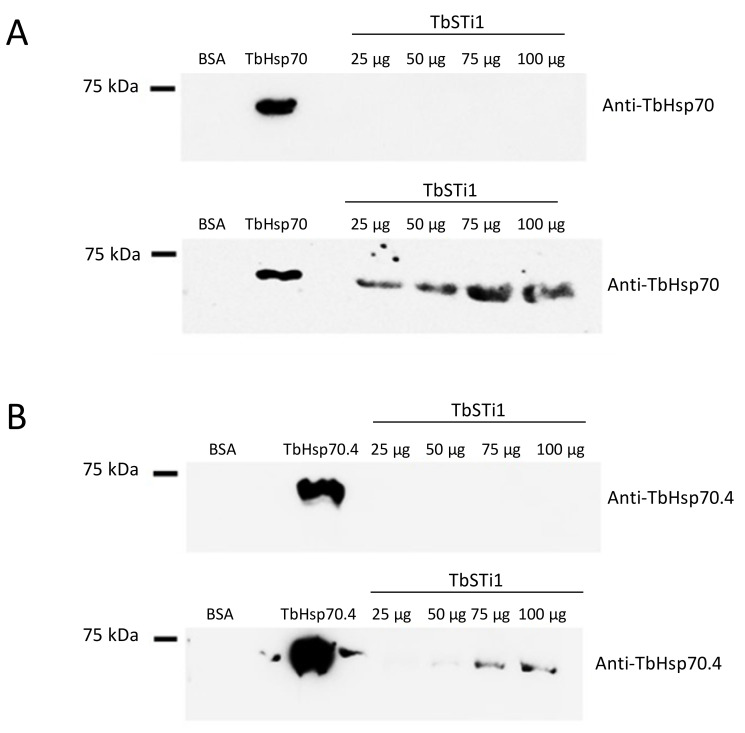
TbHsp70 and TbHsp70.4 directly interact with TbSTi1. Interaction between the TbHsp70s, TbHsp70 and TbHsp70.4 and TbSTi1 was investigated using far western analysis. TbHsp70 (50 µg); control protein, BSA (50 µg) and TbSTi1 at various concentrations (25 µg, 50 µg, 75 µg, 100 µg) were resolved by SDS-PAGE and transferred onto a blot and the blot was probed using rabbit polyclonal anti-TbHsp70.4 (**A**; top panel). A similar blot was then overlaid with TbHsp70 and it was probed using anti-TbHsp70 (**A**; bottom panel). (**B**) TbHsp70.4 protein (50 µg); control protein, BSA (50 µg) and TbSTi1 protein at various concentrations (25 µg, 50 µg, 75 µg, 100 µg) were resolved by SDS-PAGE and transferred to a blot and the blot was probed using rabbit polyclonal anti-TbHsp70.4 (**B**; top panel). A similar blot was then overlaid with TbHsp70.4 (75 µg) and it was probed using anti-TbHsp70.4 (**B**; bottom panel). The blots are representative of independent replicates.

In light of the GGMP repeat residues and C-terminal EEVD motif being absent in TbHsp70.c, we enquired if this chaperone is capable of binding to the co-chaperone TbSTi1, which is predicted to localize to the cytosol. To address this question, we employed far western analysis. As expected, we demonstrated direct interaction between TbSTi1 and a canonical Hsp70 chaperone, TbHsp70, which possesses both the GGMP repeat residues and C-terminal EEVD motif (Figure 5A). On the other hand, TbHsp70.4 possesses a divergent C-terminal DDVD motif, in place of the EEVD motif present in canonical Hsp70s, and it also lacks the GGMP repeat motif. In spite of this, TbHsp70.4 was capable of interacting with TbSTi1 *in vitro* (Figure 5B). However, the direct interaction between TbHsp70.4 and TbSTi1 was only evident at high concentrations of TbSTi1 (75 µg, 100 µg; Figure 5B). The absence of the GGMP repeat motif, as well as the presence of a divergent DDVD motif, may explain why the strength of TbHsp70.4 binding TbSTi1 is weaker than was observed for TbHsp70 binding to TbSTi1 (Figure 5).

**Figure 6:**
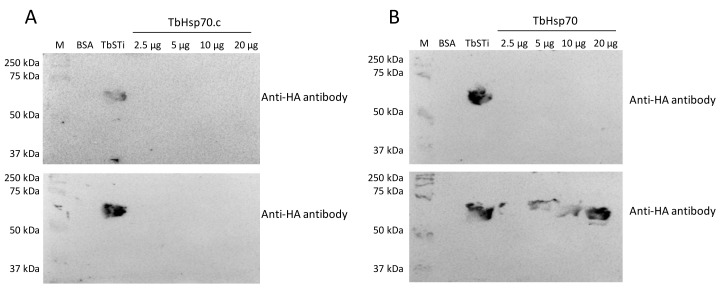
TbSTi1 interacts with TbHsp70 but not with TbHsp70.c. Far western analysis was used to investigate the interactions between TbHsp70.c (**A**) and TbHsp70 (**B**) with TbSTi1. BSA (20 µg/mL) as negative control protein, TbSTi1 (20 µg/mL) as positive control protein, and TbHsp70 or TbHsp70.c proteins at various concentrations (2.5 µg/mL, 5 µg/mL, 10 µg/mL, and 20 µg/mL) were resolved by SDS-PAGE and transferred to the respective blots that were then probed using anti-HA antibody (top panel), an epitope of TbSTi1. Similarly transferred blots were overlaid with TbSTi1 then also probed with anti-HA antibody (bottom panel). The blots are representative of independent replicates.

Interestingly, there was no evidence for interaction between TbHsp70.c and TbSTi1 (Figure 6A). This suggests that TbHsp70.c may not cooperate with *T. brucei* TbHsp83. This further suggests that TbHsp70.c is a unique cytosolic Hsp70 in *T. brucei* that does not require TbSTi to form a functional complex with TbHsp83 to facilitate the folding of its client proteins. However, further interaction studies are needed to confirm that TbHsp70.c does not form a partnership with TbSTi in the parasite. Far western analysis of the direct interaction between TbHsp70 and TbSti1 was performed as control (Figure 6B), considering that this interaction had been shown previously (Figure 5A). The recombinant TbSTi1 was expressed with an N-terminal HA tag, and, therefore, anti-HA antibodies were used to determine if TbSTi1 formed a complex with either the underlaid TbHsp70 and TbHsp70.c (Figure 6). To validate the far western data, it was important to demonstrate that the anti-TbSTi1 and the anti-HA antibodies that were employed in the assay were incapable of cross-reacting with TbHsp70 or TbHsp70.c as demonstrated (Figure 6, top panel). Similarly, it was essential to show that the anti-TbHsp70 and anti-TbHsp70.4 antibodies used in this assay do not cross-react with the TbSTi1 (Figure 5).

### 2.5. Both Quercetin and Methylene Blue Are Lethal to T. Brucei Parasites and Modulate the Activity of TbHsp70.c

**Figure 7:**
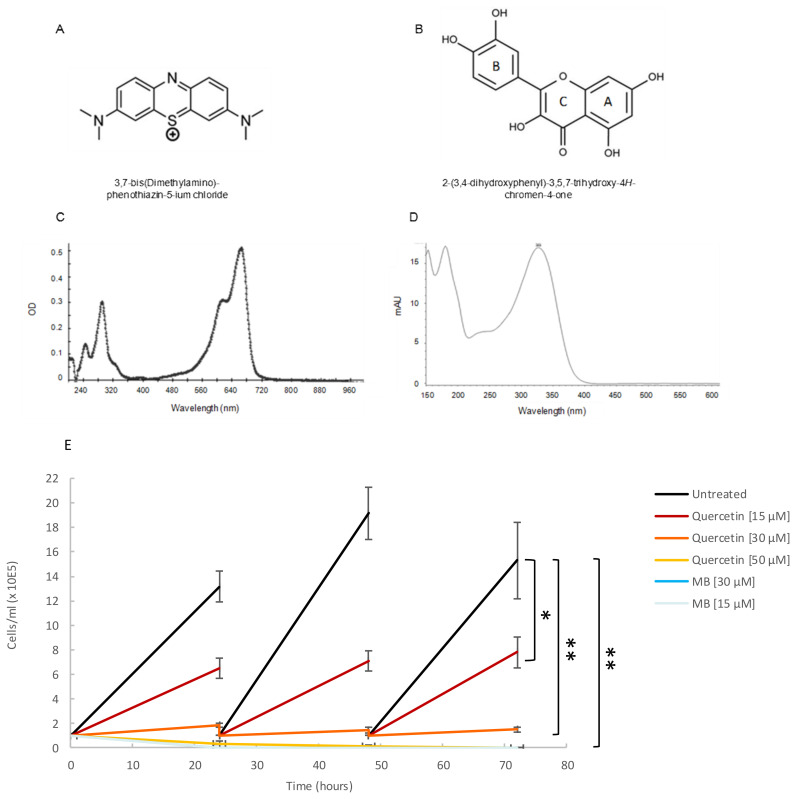
Quercetin and methylene blue inhibits growth of the bloodstream form of *T. brucei.* (**A**) Tetramethylthionine, commonly called methylene blue (MB), is a phenothiazine: a heterocyclic aromatic compound consisting of two benzene rings, each with a methylamino group, adjacent to a single thiazine. (**B**) Natural flavonoid quercetin. (**C**) A spectral scan of MB (30 µM) suspended in assay buffer revealed a UV visible spectrum at A290 nm and another at A665 nm. (**D**) A spectral scan of quercetin resulted in a UV-visible spectrum at A369 nm. (**E**) The phenotypic effect of quercetin (15 µM; 30 µM; 50 µM), and MB (15 µM; 30 µM) on *T. brucei* cells was assessed by means of growth curve analysis. The Y-axis represents 1 × 10^5^ cells/mL and the X-axis the duration of the experiment, in hours. The error bars were generated from data obtained using independent *T. brucei* cell preparations. Statistical significance was determined by using one-way ANOVA and post-hoc test (* *p* < 0.01; ** *p* < 0.001).

Based on their reported ability to inhibit either Hsp70 synthesis or the ATPase activity of Hsp70 [30], we selected two small molecule modulators to investigate their effect on the basal ATPase activity of TbHsp70.c and on the Tbj2-stimulated ATPase activity of TbHsp70.c. The structures of methylene blue (Figure 7A) and quercetin (Figure 7B) are shown. A spectral scan of methylene blue and quercetin revealed a UV-visible spectrum at A290 nm and A665 nm (Figure 7C), and at A369 nm (Figure 7D), respectively. A growth curve study showed that both quercetin (at concentrations of 15 µM, 30 µM, and 50 µM) and methylene blue (at concentrations of 15 µM and 30 µM) are toxic to bloodstream forms of *T. brucei* parasites (Figure 7E). Previous studies have shown quercetin to have anti-plasmodial activity with an IC50 value of 15 µM [31], and methylene blue was demonstrated to have gametocytocidal activity with an IC50 value of 1060.2 nM [32].

**Figure 8:**
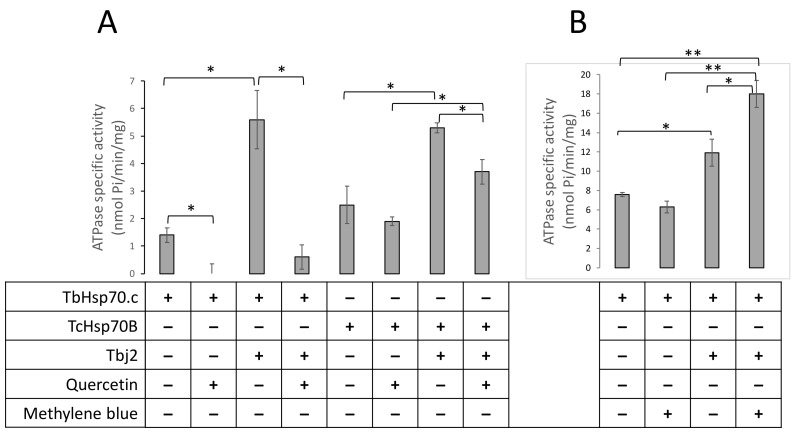
Quercetin inhibits whilst methylene blue enhances TbHsp70.c-Tbj2 activity. The effect of quercetin (**A**) and MB (**B**) on the basal and Tbj2-stimulated ATPase activity of TbHsp70.c and TcHsp70B was investigated. Inorganic phosphate released by ATP in the presence of each Hsp70 was monitored by direct calorimetry at 595 nm wavelength. TbHsp70.c, TcHsp70B, and Tbj2 concentrations were maintained at 0.4 µM, and ATP was present in all reactions at 600 µM (**A**). Quercetin was present in the reactions as indicated at 30 µM. The effect of MB on the basal ATPase activity of TbHsp70.c and TbHsp70.c in partnership with Tbj2 was investigated (**B**). TbHsp70.c and Tbj2 concentrations were kept constant at 1 µM and ATP was present in all reactions at 600 µM. MB was present at a concentration of 30 µM. The error bars indicate data generated from three assays conducted using independent TbHsp70.c, Tbj2, and TcHsp70B protein preparations. Statistical significance was determined by using one-way ANOVA and post-hoc test (* *p* < 0.01; ** *p* < 0.001).

Next, we explored the possible inhibition of TbHsp70.c by quercetin and methylene blue. Our findings suggest that quercetin inhibits the basal ATPase activity of TbHsp70.c (Figure 8A). Upon the addition of quercetin to the TbHsp70.c in the presence of Tbj2, the ATPase activity of the chaperone complex decreased from 5.6 to 0.6 nmol Pi/min/mg, a considerable 9.3-fold inhibition of Tbj2-stimulated ATPase activity of TbHsp70.c (Figure 8A). We introduced a canonical Hsp70 from *T. cruzi*, TcHsp70B, to further investigate the effect of quercetin. Tbj2 has previously been shown to stimulate the ATPase activity of TcHsp70B [22]. Our findings suggest that quercetin induced a 1.3-fold inhibition of the ATPase activity of TcHsp70B and a 1.4-fold inhibition of the Tbj2-stimulated ATPase activity (Figure 8A). Notably, TbHsp70.c-Tbj2 exhibited higher sensitivity to quercetin inhibition than was observed for TcHsp70B-Tbj2 (Figure 8A).

On the other hand, methylene blue did not significantly affect the basal ATPase activity of TbHsp70.c (activity dropped from 7.6 to 6.4 nmol Pi/min/mg; Figure 8B). Unexpectedly, in the presence of Tbj2, methylene blue enhanced the ATPase activity of TbHsp70.c (12.5 nmol Pi/min/mg to 18.7 nmol Pi/min/mg representing a 1.5-fold increase in ATPase activity; Figure 8B). This implies that the effect of methylene blue on the basal ATPase activity of TbHsp70.c is distinct from its effect on Tbj2-stimulated ATPase activity.

### 2.6. Quercetin and Methylene Blue Docks to the Nucleotide Binding Pocket of TbHsp70.c

**Figure 9:**
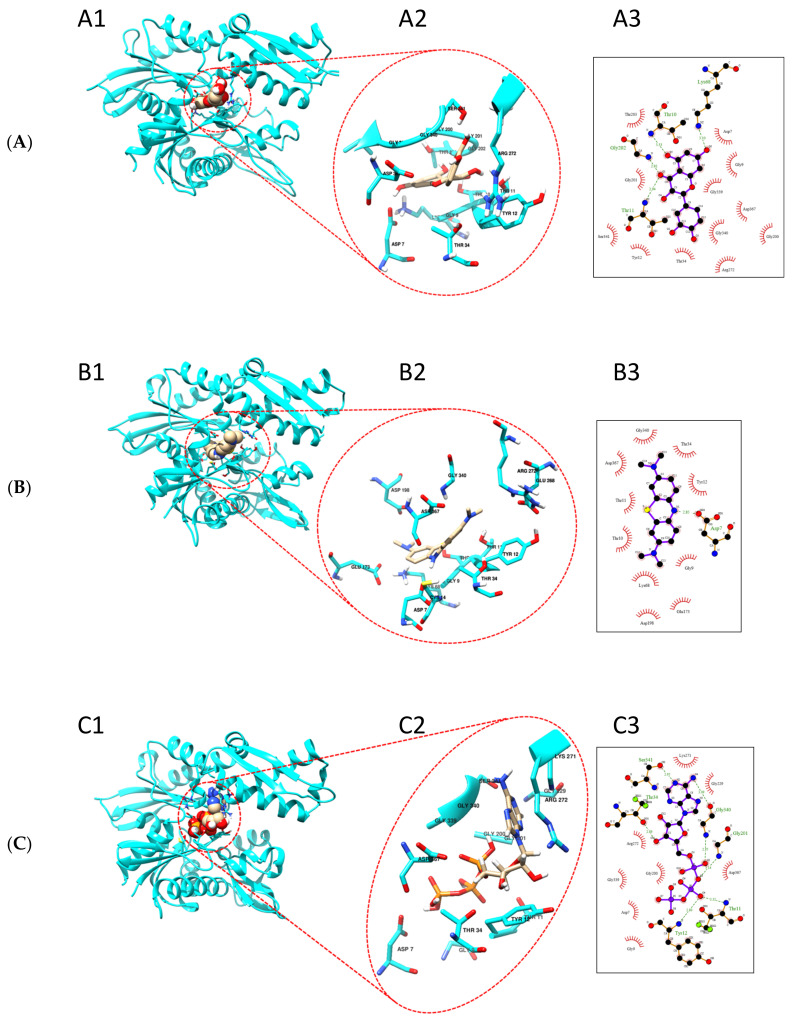
Putative binding sites of quercetin and methylene blue on TbHsp70.c. Homology model of the ATPase domain of TbHsp70.c showing predicted binding with (**A**) quercetin, (**B**) methylene blue, and (**C**) ATP. (**A1**–**C1**) show the ATPase domain of TbHsp70.c binding quercetin, methylene blue, and ATP, respectively; (**A2**–**C2**) show the zoomed-in image of the binding sites; (**A3**–**C3**) show the residues that form hydrogen bonds (represented by dotted lines), and residues that form hydrophobic interactions (represented by lash-resembling structures) with quercetin, methylene blue, and ATP, respectively.

Based on our findings that quercetin and methylene blue modulate the ATPase activity of TbHsp70.c, we further predicted the possible binding sites of these small molecule modulators. We generated a homology model of the ATPase domain of TbHsp70.c (Figure 9). We predict that quercetin binds to the nucleotide binding pocket of TbHsp70.c, forming hydrogen bonds with residues T10, T11, K68, and G202. It is further predicted to interact with TbHsp70.c via hydrophobic interactions (Figure 9A). Altogether, this interaction registers a docking score of −9.5 kcal/mol. Similarly, we predict that methylene blue binds to the nucleotide binding pocket of TbHsp70.c, forming a hydrogen bond with residue D7, and interaction with TbHsp70.c also occurs via hydrophobic interactions (Figure 9B), with a docking score of −8.3 kcal/mol. Furthermore, we predict that the binding of ATP to the nucleotide binding pocket of TbHsp70.c occurs via hydrogen bonds and hydrophobic interactions, with a docking score of −9.0 kcal/mol, which is comparable to the docking scores calculated for quercetin and methylene blue (Figure 9C).

## 3. Discussion

Hsp70s have been well established to maintain proteostasis and regulate proper folding of misfolded substrates. The stress-inducible TbHsp70.c was previously shown to be an ATPase that also functions as a holdase protein, and Tbj2 was shown to enhance the ATP-driven activity of TbHsp70.c, suggesting its possible role as co-chaperone of TbHsp70.c [22]. The current study demonstrates that TbHsp70.c exhibits unique structure-function features and that it localizes to the parasite cytosol and nucleus. Furthermore, we demonstrated that it is incapable of interacting with the co-chaperone, TbSTi1. This suggests that the chaperone function of TbHsp70.c may be independent of TbHsp83. However, it should be noted that the assay demonstrating lack of interaction between TbHsp70.c and TbSTi1 was based on in vitro analysis and this phenomenon remains to be validated in vivo. Our findings further suggest that both methylene blue and quercetin may target the function of TbHsp70.c to inhibit *T. brucei* growth.

Notably, TbHsp70.c lacks the GGMP repeat residues and the C-terminal EEVD residues implicated in substrate binding and co-chaperone recognition [17]. In this study, TbSTi1 did not interact with TbHsp70.c; however, there was evidence of a stronger association between TbHsp70 and TbSTi1 than between TbHsp70.4 and TbSTi1. This is possibly due to the fact that TbHsp70.4 possesses a C-terminal DDVD motif in place of the EEVD motif present in canonical cytosolic Hsp70s. Furthermore, TbHsp70.4 lacks the GGMP motif implicated in STi1 binding. The TPR domains of STi1 are highly charged suggesting that their interaction with Hsp70 is electrostatic [33]. Thus, the substitution of the glutamic acid for aspartic acid residues in the C-terminal DDVD motif of TbHsp70.4 may account for weaker electrostatic interactions with the carboxylate clamp residues defined by the TPR1 domain of STi1. However, this must be conclusively elucidated using quantitative biophysical interaction techniques such as surface plasmon resonance analysis. While TbHsp70 has been shown previously to be heat inducible, TbHsp70.4 is constitutively expressed [11]. On the other hand, TbHsp70.c also appears to be heat inducible [22]. This suggests that under stressful conditions TbHsp70.c may complement the function of TbHsp70 as both are stress inducible. However, their structural variation suggests that they conduct specialized functions under the stressful conditions.

Based on amino acid sequence, TbHsp70.c is functionally distinct from the canonical Hsp70s. This makes it an interesting protein with respect to its prospects as an anti-trypanosomal drug target. Indeed, we demonstrated that TbHsp70.c basal ATPase and Tbj2-stimulated ATPase activities are modulated by methylene blue and quercetin. While these findings may be inadequate to explain whether TbHsp70.c is the sole candidate targeted by quercetin and methylene blue to inhibit parasite growth, their prospects suggest that TbHsp70.c may hold promise as a druggable candidate. It should be noted that even though methylene blue exhibits trypanocidal activity in vitro, a previous study showed that it is therapeutically ineffective based on a mouse model [34]. A previous study reported that methylene blue inhibited the ATPase activity of human Hsp70 by at least 80% [35]. This is worrying as it apparently suggests that methylene blue may have adverse effects on human Hsp70 function. A study to validate the comparative effects of methylene blue on human Hsp70 and TbHsp70.c could be conducted in synchronized fashion; however, a rational drug design approach based on the atypical nature of TbHsp70.c may lead towards development of more successful trypanotoxic compounds.

Apart from lack of the GGMP repeats and the C-terminal EEVD motif, the first four residues (RTAG) of the linker of TbHsp70.c are unique from those present in the canonical *E. coli* DnaK (DVKD). Given the importance of the linker motif in Hsp70 function, the sequence variation we noted in the linker of TbHsp70.c could signify the functional uniqueness of this chaperone. Further highlighting its functional specificity is the presence of unique arch residues, D412 and V437 of TbHsp70.c, that substitute for residues A406 and Y431 in HSPA1A. Furthermore, residues V409 and Y434 of TbHsp70.c, implicated in substrate binding, are not conserved (Figure 4). Possibly, this divergence in the substrate binding domain mirrors the unique range of substrate/clients that TbHsp70.c folds. Since TbHsp70.c is essential in parasite differentiation [26], it may selectively target those proteins which aid in that process. The substrate binding domain of *Plasmodium falciparum* Hsp110 was shown to be divergent from that of canonical Hsp110s to contend with the asparagine-rich proteome in *P. falciparum* [36]. Therefore, the divergent substrate binding cavity of TbHsp70.c highlights that it is functionally specialized.

## 4. Materials and Methods

### 4.1. Materials

Reagents used were purchased from Sigma Chemicals Co. (St. Louis, MO, USA), Merck Chemicals (Darmstadt, Germany), BioRad (Hercules, CA, USA), or Roche Molecular Biochemicals (Indianapolis, IN, USA). Nickel beads were purchased from Pharmacia Biotech (Uppsala, Sweden). Anti-His antibody was purchased from Amersham Pharmacia Biotech (Amersham, UK). Production of anti-TbHsp70.c antibody was previously described [22]. Plasmid TcHsp70B was a gift from Dr. D. Engman (Northwestern University Medical School, Chicago, USA). The *T. brucei* TREU927 strain was a kind donation from Professor George Cross (Rockefeller University, New York, NY, USA).

### 4.2. T. brucei Culture

The *T. b. brucei* 427 Lister bloodstream form strain variant 221 was used for subcellular localization experiments. The wild type Lister 927 variant 221 *T. brucei brucei* strain trypanosomes were cultured as previously described [22]. SMB cells were treated with 1 mg/L neomycin (G418) to maintain the selective pressure. Cells were maintained at a density below 2 × 10^6^ cells/mL to avoid the slender bloodstream form cells from differentiating into the stumpy form cells [37]. Growth was measured using a Neubauer hemocytometer, after which cells were diluted, according to their density, in medium pre-warmed to 37 °C.

### 4.3. Investigation of the Subcellular Localization of TbHsp70.c Using Anti-TbHsp70.c Peptide Antibodies

Prior to culturing cells, coverslips were coated with poly-L-Lysine (70–150 kDa). Coverslips were washed in absolute EtOH overnight and 5 rinses in dH_2_O were performed before the etching in 50% HNO_3_ for 12 h at room temperature. The coverslips were repeatedly rinsed in dH_2_O for 30 min, after which they were sterilized with absolute ethanol. The coverslips were allowed to dry in a 24-well plate, after which sterile poly-L-Lysine (0.1 mg/mL) was added to each well, enough to coat, and incubated at 37 °C for 24 h. Coverslips were dried, and re-coated with poly-L-Lysine, repeating the process. Coverslips were placed in a 24-well culture plate. *T. b. brucei* 427 v.221 cells (30 mL) were cultured to a density of ~1.5 × 10^6^ cells/mL. Cells were pelleted (800 g, 10 min) and all but a little (~1.5 mL) of the medium was poured off; a half-volume PBS:FBS (1:1) was added to the resuspended cells. The cell suspension was aliquoted (200 µL) into a 24-well culture plate containing poly-L-lysine-coated coverslips and incubated at room temperature for 15 min. 1% formaldehyde/PBS (1:1) (50 µL) was added to each well to fix the cells, and incubated at 4 °C overnight. The medium was removed after which the cells were permeabilized by the addition of 200 µL 0.5% Triton X-100 in PBS/FBS (1:1) for 30 min at room temperature. Upon removing Triton X-100, rabbit anti-TbHsp70.c peptide antibody, diluted 1:1000 in 25% FBS/PBS, was added and incubated for 1 h at room temperature. The cells were washed twice with 25% FBS/PBS, each with 10-min incubations. Cells were allowed to incubate for 1 h in light-sensitive anti-rabbit Alexa Fluor^®^ 488 Dye (Invitrogen, A11008) (excitation maximum is 495 nm and emission maximum is 519 nm), diluted 1:1000 in 25% FBS/PBS, followed by three washes as before. Propidium iodide (1 mg/mL) (excitation maximum is 535 nm and emission maximum is 617 nm) was used as a nuclear counterstain, diluted 1:10 in 25% FBS/PBS, was added to the last wash. The coverslips were mounted on slides using Dako fluorescent mounting medium (DAKO, S5023), allowed to set overnight and sealed with clear nail varnish. The fluorescing cells were viewed under oil immersion with a fluorescence microscope (100x objective) with excitation at different wavelengths of light. In addition to determining the localization of TbHsp70.c, cells were analysed for their stage of the cell division cycle, observing either the possession of 1 nucleus: 1 kinetoplast (1N:1K), 1N:2K, or 2N:2K.

### 4.4. Determination of Distinct Features of TbHsp70.c

Prior to protein sequence analysis using a multiple sequence alignment, trypanosomatid amino acid sequences were obtained from the TritrypDB, and other Hsp70 protein sequences were retrieved from NCBI in FASTA format. Multiple sequence alignments were generated using ClustalW v2.0 and Boxshade v3.21. The TbHsp70.c protein sequence was used as query in a BLAST search to identify the best possible template sequence for homology modelling. Bovine Hsc70 was selected as it shared the highest sequence identity with TbHsp70.c. The template sequence and crystal structure of bovine Hsc70 (1YUW.pdb) [38] was obtained from the PDB database (http://www.rcsb.org/pdb/home/home.do; accessed on 18 May 2011) and aligned with TbHsp70.c using the ClustalW-based alignment option. The alignment was saved in a PIR output format and used to construct 100 homology models using Modeller 9v3 software [39]. The resulting PDB files were viewed using PyMol [29]. The model was validated through a series of checks including calculation of the root mean square deviation (RMSD) using PyMol v1.4, the DOPE (discrete optimized protein energy) Z-score using online software ProSA-web (https://prosa.services.came.sbg.ac.at; accessed on 2 January 2013) [40], and global distance test total score using the MetaMQAPII server (https://genesilico.pl/toolkit/unimod?method=MetaMQAPI; accessed on 2 January 2013).

### 4.5. Evaluation of the Interaction between the Cytosolic TbHsp70 Proteins and TbSTi1

The full-length *E. coli* codon-optimized sequence of *TBSTI1* (TriTrypDB accession number: Tb927.5.2190) was synthesized and supplied by the GenScript Corporation (Piscataway, NJ, USA) and inserted into the pQE60 (Qiagen, Germantown, MD, USA) between the *Bam*HI and *Hind*III restriction sites to generate the pQE60-TbSTi expression plasmid with an N-terminal HA epitope. The TbHsp70, TbHsp70.4, and TbHsp70.c constructs have been previously described. [11,22]. Far western analysis was conducted as previously described [19,41], with modifications, to evaluate the interaction of the TbSTi1 with the three TbHsp70 proteins. Briefly, for the TbHsp70.c and TbHsp70 experimental set, varying amounts (2,5–20 µg/mL) of TbHsp70.c and TbHsp70 proteins, 50 µg/mL of the ligand protein (TbSTi1), and 50 µg/mL of BSA (negative control) were resolved using 10% SDS-PAGE, while for the TbHsp70 and TbHsp70.4 set, varying amounts (25–100 µg/mL) of the TbSTi1 protein, 50 µg/mL of the ligand protein (TbHsp70 or TbHsp70.4), and 50 µg/mL of BSA (negative control) were equally resolved. The proteins were transferred onto nitrocellulose membrane (Bio-Rad, Hercules, CA, USA) and blocked in 5% (*w*/*v*) fat-free milk powder in 1x Tris-buffered saline (TBS; 50 mM Tris-HCl, pH 7.5, 150 mM NaCl) for 1 h at room temperature. The membrane was incubated with the ligand protein (75 µg/mL of either TbSTi1, TbHsp70 or TbHsp70.4) in protein binding buffer (20 mM Tris-HCl, pH 7.6, 100 mM NaCl, 0.5 mM EDTA, 10% glycerol, 0.1% Tween-20, 5% fat-free milk powder and 1 mM DTT) overnight at 4 °C with gentle agitation. For each set a control blot was incubated in binding buffer lacking ligand. After washing three times in 1x TBS-Tween buffer (0.1% Tween-20 in 1X Tris-buffered saline pH 7.5), the blots were incubated with the primary antibody ((1:10,000) anti-HA (1:10,000) for TbSTi and rabbit polyclonal anti-TbHsp70 (1:2500) and rabbit polyclonal anti-TbHsp70.4 (1:2500) antibodies for TbHsp70 and TbHsp70.4, respectively) for 2 h at 4 °C with gentle agitation. After subsequent washing steps with 1x TBS-T buffer, the membranes were incubated with goat raised anti-rabbit horseradish peroxidase conjugated secondary antibody (1:2500) for 2 h at 4 °C with gentle agitation. The membrane was washed twice using 1x TBS-T buffer to remove unbound secondary antibodies. Visualization of the protein bands on the Western blot was conducted using the ClarityTM Western ECL Blotting kit (Bio-Rad, Hercules, CA, USA) as per the supplier’s instructions, and the images were captured using the ChemiDoc Imaging system (Bio-Rad, Hercules, CA, USA).

### 4.6. Analysis of Quercetin and Methylene Blue on Parasite Growth

Trypanosomes were seeded at an initial density of 1 × 10^5^ cells/mL, using a Neubauer hemocytometer to count parasites. Parasites were subcultured every 24 h for a duration of three days in the absence or presence of quercetin (15 µM; 30 µM; 50 µM) and methylene blue (15 µM; 30 µM). The selected concentrations for inhibitors were based on a previous study [34]. Data was obtained using three independent *T. brucei* cell preparations and used to plot a growth curve using Microsoft Excel. Statistical significance was determined by using one-way ANOVA and a post-hoc test (* *p* < 0.01; ** *p* < 0.001).

### 4.7. Analysis of Quercetin and Methylene Blue on TbHsp70.c-Tbj2 Chaperone Activity

We previously demonstrated Tbj2’s ability to stimulate the ATPase activity of TbHsp70.c [22], and so we wished to determine whether reported inhibitors could affect this function. This study investigated the effects of small molecule modulators, quercetin and methylene blue, on the Tbj2-stimualted ATPase activity of TbHsp70.c. Expression and purification of the recombinant proteins were conducted as previously described [22]. A spectral scan of quercetin and methylene was conducted using a KC Junior microplate reader to ensure that the compounds were not detected at A850 nm (Figure 7). The ATPase assay was performed as previously described [22]. The protein concentrations of TbHsp70.c, TcHsp70B, and Tbj2 were maintained at 0.4 µM and quercetin was added to the reaction prior to equilibration at 37 °C to a final concentration of 30 µM. To investigate the effect of methylene blue on the activity of TbHsp70.c and Tbj2, protein concentrations were maintained at 1 µM, and MB (30 µM), suspended in ATPase buffer, was added to the reaction prior to equilibration at 37 °C.

### 4.8. Quercetin and Methylene Blue Are Predicted to Bind the ATPase Domain of TbHsp70.c

Amino acid sequences of *T. brucei* Hsp70c ATPase domain (Accession: Tb927.11.11290) were retrieved from UniprotKB and supplied as a query for protein BLAST [42] against the SWISS-MODEL template library to generate a model using SWISS-MODEL webserver (https://swissmodel.expasy.org/interactive; accessed on 21 April 2021) [43]. Tetramethylthionine (methylene blue) and 3,3′,4′,5,7-Pentahydroxyflavone (Quercetin) 2D structures were retrieved from PubChem [44]. The structures were inspected for valid bond orders, energy minimized using the General Amber Force Field (GAFF) implemented in Avogadro v2.0 [45] software, and saved in a 3D mol2 format. AutoDock Vina [46] was used to conduct molecular docking calculations. The Gasteiger partial charges and AutoDock atom types were assigned using AutoDock user interface provided by MGL Tools v1.5.6. A blind docking strategy was used to predict ligand binding site on a protein. A grid box was generated with the following grid coordinates x = 25.004 Å, y = 0.644 Å, and z = 5.395 Å for the centre and x = 62 Å, y = 70 Å, and z = 68 Å for dimensions and with exhaustiveness = 8. A spacing of 0.925 Å was used to centralize the grid box on the protein. The generated grid box encapsulated the whole protein surface for sufficient binding site identification. A Lamarckian algorithm was applied to generate docked ligand conformations of docking scores arranged in a decreasing manner. Ligand conformations with the best docking score and RMSD = 0.0 Å were analysed and complexed to the protein structure using UCSF Chimera [47] and saved in pdb format for further analysis.

## 5. Conclusions

Currently, there are limited effective treatment strategies against African trypanosomiasis, and this warrants the need to identify *T. brucei* druggable protein candidates and develop more treatment options. Our current findings highlight TbHsp70.c as a unique chaperone that localizes to the cytosol and to the nucleus. Its localization to the cytosol suggests that it could be a custodian of several proteins that occur in this compartment, and this could explain why it is essential for parasite growth. It remains to be established what role TbHsp70.c plays in the nucleus and whether it plays a role in DNA repair and replication. We further demonstrated that methylene blue and quercetin could modulate TbHsp70.c activity suggesting that targeting this atypical chaperone hold prospects in the fight against *T. brucei*. However, it is possible that methylene blue and quercetin may target other Hsp70s of the parasite besides TbHsp70.c. Nonetheless, the current work paves the way for further studies targeting the role of TbHsp70.c towards possible intervention against human African trypanosomiasis.

## Data Availability

Data is contained within the article or Supplementary Materials.

## Supplementary Materials

The following are available online at https://www.mdpi.com/article/10.3390/ijms22136776/s1.
