# Supplementary material for: Characterization of an Atypical Trypanosoma brucei Hsp70 Demonstrates Its Cytosolic-Nuclear Localization and Modulation by Quercetin and Methylene Blue
Source: Int J Mol Sci. 2021 Jun 24;22(13):6776. doi: 10.3390/ijms22136776 (PMC8269394; doi:10.3390/ijms22136776)
Supplement: Supplementary file 1 [file ijms-22-06776-s001.zip › ijms-1218076-supplementary.pdf]

# SUPPLEMENTARY MATERIALS

```
EcDnaK        1 -------------MGKIIGIDLGTTNSCVAIMDGTTPRVIENAEGDRTTPSIIAYTQDGE
TbHsp70.c     1 -----------MTYEGAIGIDLGTTYSCVGVWQNERVEIIANDQGNRTTPSYVAFV-NNE
TbHsp70.4     1 ------------MPAPAIGIDLGTTYSCVGVFKNDQVEIVANDQGNRTTPSYVSFS-ETE
TcHsp70B      1 -----------MTYEGAIGIDLGTTYSCVGVWQNERVEIIANDQGSRTTPSYVAFT-DTE
TbHsp70       1 -----------MTYEGAIGIDLGTTYSCVGVWQNERVEIIANDQGNRTTPSYVAFT-DSE
PfHsp70-1     1 MASAKGSKPNLPESNIAIGIDLGTTYSCVGVWRNENVDIIANDQGNRTTPSYVAFT-DTE
HsHSPA6       1 ---------MQAPRELAVGIDLGTTYSCVGVFQQGRVEILANDQGNRTTPSYVAFT-DTE
HsHSPA1A      1 -----------MAKAAAIGIDLGTTYSCVGVFQHGKVEIIANDQGNRTTPSYVAFT-DTE
BtHsc70       1 -----------MSKGPAVGIDLGTTYSCVGVFQHGKVEIIANDQGNRTTPSYVAFT-DTE
                           .    ..*******.***............*..*.*****..... ..*

EcDnaK       48 TLVGQPAKRQAVTNPQNTLFAIKRLIGRRFQDEEVQRDVSIMPFKIIAADNGDAWVEVK-
TbHsp70.c    49 VLVGDAAKNHAARGSNGVIFDAKRLIGRKFSDSVVQSDMKHWPFKVEEGEKGGAVMRVEH
TbHsp70.4    48 RLVGDAAKNQVAMNPTNTVFDAKRIIGRKYDDPDLQADMKHWPFKVTVK-EGKPVVEVEY
TcHsp70B     49 RLIGDAAKNQVAMNPTNTVFDAKRLIGRKFSDPVVQSDMKHWPFKVITKGDDKPVIQVQF
TbHsp70      49 RLIGDAAKNQVAMNPTNTVFDAKRLIGRKFSDSVVQSDMKHWPFKVVTKGDDKPVIQVQF
PfHsp70-1    60 RLIGDAAKNQVARNPENTVFDAKRLIGRKFTESSVQSDMKHWPFTVKSGVDEKPMIEVTY
HsHSPA6      51 RLVGDAAKSQAALNPHNTVFDAKRLIGRKFADTTVQSDMKHWPFRVVSE-GGKPKVRVCY
HsHSPA1A     49 RLIGDAAKNQVALNPQNTVFDAKRLIGRKFGDPVVQSDMKHWPFQVIND-GDKPKVQVSY
BtHsc70      49 RLIGDAAKNQVAMNPTNTVFDAKRLIGRRFDDAVVQSDMKHWPFMVVND-AGRPKVQVEY
                .*.*..**....... ...*..**.***.. . ..*.*....**...    ..... * .

EcDnaK      107 ---GQKMAPPQISAEVLKKMKKTAEDYLGEPVTEAVITVPAYFNDAQRQATKDAGRIAGL
TbHsp70.c   109 LGEGMLLQPEQISARVLAYLKSCAESYLGKQVAKAVVTVPAYFNDSQRQATKDAGTIAGL
TbHsp70.4   107 QGERRTFFPEEISAMVLQKMKEIAESYLGEKVSKAVVTVPAYFNDSQRQATKDAGSIAGL
TcHsp70B    109 RGETKTFNPEEVSSMVLSKMKEIAESYLGKQVKKAVVTVPAYFNDSQRQATKDAGTIAGM
TbHsp70     109 RGETKTFNPEEISSMVLLKMKEVAESYLGKQVAKAVVTVPAYFNDSQRQATKDAGTIAGL
PfHsp70-1   120 QGEKKLFHPEEISSMVLQKMKENAEAFLGKSIKNAVITVPAYFNDSQRQATKDAGTIAGL
HsHSPA6     110 RGEDKTFYPEEISSMVLSKMKETAEAYLGQPVKHAVITVPAYFNDSQRQATKDAGAIAGL
HsHSPA1A    108 KGDTKAFYPEEISSMVLTKMKEIAEAYLGYPVTNAVITVPAYFNDSQRQATKDAGVIAGL
BtHsc70     108 KGETKSFYPEEVSSMVLTKMKEIAEAYLGKTVTNAVVTVPAYFNDSQRQATKDAGTIAGL
                ... ... *...*..** ..*..** .**. . .**.********.*********.***.

EcDnaK      164 EVKRIINEPTAAALAYGLDKGT--GNRTIAVYDLGGGTFDISIIEIDEVDGEKTFEVLAT
TbHsp70.c   169 EVLRIINEPTAAAIAYGLDKADEGKERNVLVFDFGGGTFDVSIISVSG----GVFEVKAT
TbHsp70.4   167 EVLRIVNEPTAAAIAYGMDRSSEGAMKTVLIFDLGGGTFDVTLLNIDG----GLFEVRAT
TcHsp70B    169 EVLRIINEPTAAAIAYGLDKVEDGKERNVLIFDLGGGTFDVTLLTIDG----GIFEVKAT
TbHsp70     169 EVLRIINEPTAAAIAYGLDKADEGKERNVLIFDLGGGTFDVTLLTIDG----GIFEVKAT
PfHsp70-1   180 NVMRIINEPTAAAIAYGLHKKGK-GEKNILIFDLGGGTFDVSLLTIED----GIFEVKAT
HsHSPA6     170 NVLRIINEPTAAAIAYGLDRRGA-GERNVLIFDLGGGTFDVSVLSIDA----GVFEVKAT
HsHSPA1A    168 NVLRIINEPTAAAIAYGLDRTGK-GERNVLIFDLGGGTFDVSILTIDD----GIFEVKAT
BtHsc70     168 NVLRIINEPTAATIAYGLDKKVG-AERNVLIFDLGGGTFDVSILTIED----GIFEVKST
                .*.**.******..***...    ........*.******........    ..***..*

EcDnaK      222 NGDTHLGGEDFDSRLINYLVEEFKKDQ-GIDLRNDPLAMQRLKEAAEKAKIELSSAQQTD
TbHsp70.c   225 NGDTHLGGEDVDAALLEHALADIRNRYGIEQGSLSQKMLSKLRSRCEEVKRVLSHSTVGE
TbHsp70.4   223 AGDTHLGGEDFDSRLVDYFATEFRTRT-GKDLRGNARAMRRLRTACERVKRTLSSSASTN
TcHsp70B    225 NGDTHLGGEDFDNRLVSHFTDEFKRKNKGKDLTTSQRALRRLRTACERAKRTLSSAAQAT
TbHsp70     225 NGDTHLGGEDFDNRLVAHFTEEFKRKNKGKDLSSNLRALRRLRTACERAKRTLSSAAQAT
PfHsp70-1   235 AGDTHLGGEDFDNRLVNFCVEDFKRKNRGKDLSKNSRALRRLRTQCERAKRTLSSSTQAT
HsHSPA6     225 AGDTHLGGEDFDNRLVNHFMEEFRRKH-GKDLSGNKRALRRLRTACERAKRTLSSSTQAT
HsHSPA1A    223 AGDTHLGGEDFDNRLVNHFVEEFKRKH-KKDISQNKRAVRRLRTACERAKRTLSSSTQAS
BtHsc70     223 AGDTHLGGEDFDNRMVNHFIAEFKRKH-KKDISENKRAVRRLRTACERAKRTLSSSTQAS
                .*********.*..............  ..... . .....*....*..*..**......

EcDnaK      281 VNLPYITADATGPKHMNIKVTRAKLESLVEDLVNRSIEPLKVALQDAGLSVSDIDDVILV
TbHsp70.c   285 IALDGLLPD---GEEYVLKLTRARLEELCTKIFARCLSVVQRALKDASMKVEDIEDVVLV
TbHsp70.4   282 IEIDALY-E---GFDFFSKITRARFEEMCRDQFERCLEPVRKVLKDAEVDASAVDDVVLV
TcHsp70B    285 IEIDALF-D---NVDFQATITRARFEELCGDLFRGTLQPVERVLQDAKMDKRAVHDVVLV
TbHsp70     285 IEIDALF-E---NIDFQATITRARFEELCGDLFRGTLQPVERVLQDAKMDKRAVHDVVLV
PfHsp70-1   295 IEIDSLF-E---GIDYSVTVSRARFEELCIDYFRDTLIPVEKVLKDAMMDKKSVHEVVLV
HsHSPA6     284 LEIDSLF-E---GVDFYTSITRARFEELCSDLFRSTLEPVEKALRDAKLDKAQIHDVVLV
HsHSPA1A    282 LEIDSLF-E---GIDFYTSITRARFEELCSDLFRSTLEPVEKALRDAKLDKAQIHDLVLV
BtHsc70     282 IEIDSLY-E---GIDFYTSITRARFEELNADLFRGTLDPVEKALRDAKLDKSQIHDIVLV
                .... .. .   ....  ...**..*... .... ........*.**....  .....**
```

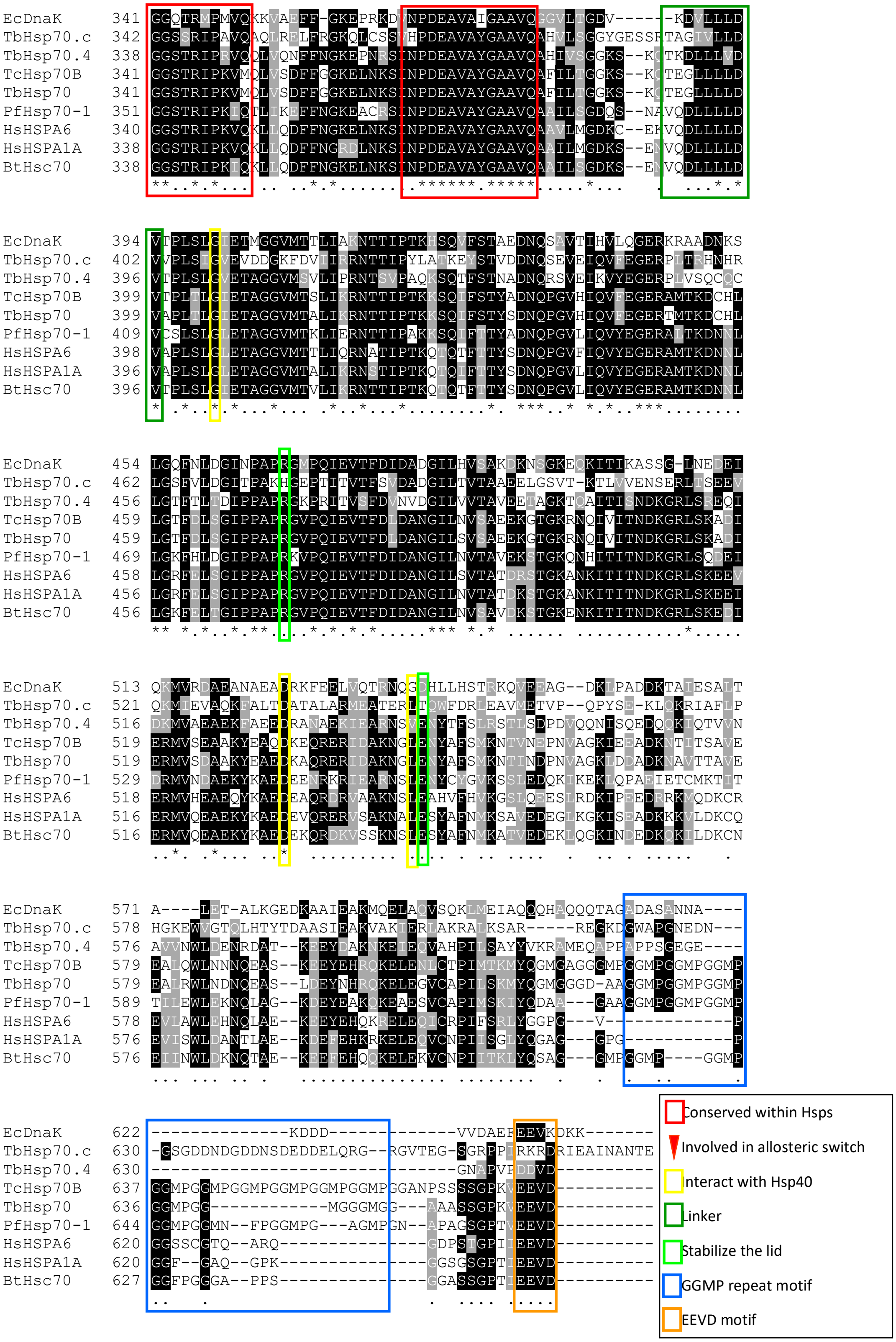


**Figure S1. Multiple sequence alignment of predicted cytosolic Hsp70 proteins from *T. brucei* and eukaryotic and prokaryotic canonical Hsp70s.**

Protein sequences for TbHsp70.c and it homologues and orthologues were retrieved from TriTrypDB and NCBI and their accession numbers are given in brackets: HsHSPA1A (AQY76873.1); HsHSPA6 (NP_002146.2); BtHsc70 (NP_776770.2); PfHsp70-1 (PF3D7_0818900); *EcDnaK* - *E. coli* DnaK (NP_414555.1); TbHsp70 (Tb927.11.11330); TcHsp70B (Tc00.1047053511211.170); TbHsp70.4 (Tb927.7.710); TbHsp70.c (Tb927.11.11290). ClustalW and Boxshade (http://www.ch.embnet.org/software/BOX_form.html) software was used to generate this alignment. Identical residues are white against a black background, and similar residues are against a grey background.
